# Preclinical Evaluation of* In Vitro* and* In Vivo* Antiviral Activities of KCT-01, a New Herbal Formula against Hepatitis B Virus

**DOI:** 10.1155/2018/1073509

**Published:** 2018-07-04

**Authors:** Hong Kim, Eungyeong Jang, So-Young Kim, Ji-Yoon Choi, Na-Rae Lee, Dae-Sung Kim, Kyung-Tae Lee, Kyung-Soo Inn, Bum-Joon Kim, Jang-Hoon Lee

**Affiliations:** ^1^Department of Microbiology and Immunology, Liver Research Institute, Cancer Research Institute and SNUMRC, College of Medicine, Seoul National University, 103 Daehak-ro, Jongno-gu, Seoul 03080, Republic of Korea; ^2^Department of Internal Medicine, College of Korean Medicine, Kyung Hee University, 26 Kyungheedae-ro, Dongdaemun-gu, Seoul 02447, Republic of Korea; ^3^Department of Internal Medicine, Kyung Hee University Korean Medicine Hospital, 23 Kyungheedae-ro, Dongdaemun-gu, Seoul 02447, Republic of Korea; ^4^Department of Fundamental Pharmaceutical Science, Graduate School, Kyung Hee University, 26 Kyungheedae-ro, Dongdaemun-gu, Seoul 02447, Republic of Korea; ^5^Hanpoon Pharmacy Company Limited, 301, Wanjusandan 6-ro, Bongdong-eup, Wanju Gun, Jeollabuk-do 55316, Republic of Korea; ^6^Department of Pharmaceutical Biochemistry, College of Pharmacy, Kyung Hee University, 26 Kyungheedae-ro, Dongdaemun-gu, Seoul 02447, Republic of Korea; ^7^Department of Life and Nanopharmaceutical Science, College of Pharmacy, Kyung Hee University, 26 Kyungheedae-ro, Dongdaemun-gu, Seoul 02447, Republic of Korea

## Abstract

Hepatitis B virus (HBV) infectious diseases currently remain incurable due to limitations of conventional antivirals such as incapability of eradicating HBV DNA, prolonged use, drug resistance, and virological relapse. KCT-01, a 30% ethanol extract consisting of* Artemisia capillaris*,* Sanguisorba officinalis*, and* Curcuma longa*, was newly developed. The objective of this study was to investigate pharmacological activities of KCT-01 against HBV using HepG2.2.15 cells and a hydrodynamic injection model. KCT-01 significantly lowered antigen secretion, virion production, and pgRNA synthesis in HepG2.2.15 cells without affecting cell viability. KCT-01 administration also resulted in significant decrease of serum virion production, liver covalently closed circular (ccc) DNA levels, and mRNA synthesis of cytokines in the liver of mice injected with HBV DNA hydrodynamically. Interestingly, coadministration of KCT-01 with entecavir enhanced its* in vitro* and* in vivo* antiviral activities. Moreover, safety of KCT-01 was assured up to 5000 mg/kg in rats in both single and repeated-dose preclinical studies. Taken together, our findings demonstrate that KCT-01 is capable of suppressing HBV replication and inflammatory cytokine production in* in vitro* and* in vivo* models without showing toxicity, suggesting the potential of using KCT-01 alone or in combination with entecavir as antiviral agent.

## 1. Introduction

Hepatitis B virus (HBV), a major pathogen that causes chronic infection of the liver, is a serious global health burden [1]. Approximately 350 million people are suffering from chronic hepatitis B (CHB) worldwide. It has been estimated that 500,000 to 1,000,000 people die of HBV-related liver diseases annually [2]. Approximately 15-40% of CHB patients may develop exacerbated liver conditions such as cirrhosis, liver failure, and hepatocellular carcinoma (HCC) [3]. Current treatment of CHB depends on several oral nucleos(t)ide analogs (lamivudine, adefovir, telbivudine, entecavir (ETV), and tenofovir) and interferon therapy [4]. Although these therapeutic strategies over the past decade have been very potent in suppressing viral replication, 30-76% of patients treated with lamivudine or adefovir have shown drug resistance and virological breakthrough due to viral mutation after long-term use for 5 years or more. ETV or tenofovir has yet to be definitely investigated on its relevance with renal toxicity or undesirable side effects after long-term oral administration [5]. These shortcomings of current conventional anti-HBV agents expose patients with CHB to a high risk of developing liver cirrhotic or hepatocarcinogenic change [6].

A variety of herbal medicines associated with treatment of liver diseases have been used as therapeutic alternatives for a long time. In China, 80% of patients with CHB receive herbal medicines [7]. It has been reported that herbal medicines generally have lower toxicity with better antiviral effects compared to lamivudine or interferon therapy [8]. In Korea,* Injinchunggan-tang* (IJCGT), a modified herbal decoction composed of 11 different medicinal herbs, has been prescribed for patients with viral hepatitis, liver cirrhosis, and HCC since the late 1990s. It has been revealed that* Artemisia capillaris* Thunberg (*A. capillaris*), a representative agent of IJCGT, can improve a wide spectrum of liver diseases, ranging from nonalcoholic steatohepatitis to HCC [9].* Sanguisorba officinalis* Linné (*S. officinalis*) has been found to have potential antiviral effects against HBV [10–12].* Curcuma longa* Linné (*C. longa*) has been used as traditional medicine for diverse diseases.* C. longa* and curcumin, its main constituents, have been shown to be able to inhibit the activation of hepatostellate cells and ameliorate liver fibrosis progression [13]. After extensive review and some basic screening tests to determine HBsAg secretion suppressive activities of herbal medicines, these three herbal medicines (*A. capillaris*,* S. officinalis*, and* C. longa*) were selected to produce a novel decoction KCT-01 to suppress HBV replication and HBV-related inflammatory responses in liver.

Due to limited host range of HBV infection,* in vivo* surrogate models are required to study HBV infectious liver diseases [14]. In particular, chimpanzees are the only known in vivo models adequate for investigating virologic response and the progression of HBV-related liver diseases [15, 16]. High expense, animal ethics, and availability difficulty of chimpanzees motivate the development of small mice models. However, there still exist challenges for inducing inflammatory cytokines and HBV infection in mice. Previous studies have demonstrated that HBV PreS1 mutation (W4P) plays a key role in the progression of CHB into cirrhosis or HCC. It contributes to male predominance in the prevalence of HCC. In addition, W4P mutations increase production of IL-6, an inflammatory cytokine critical for tumorigenesis. Male mice injected with HBV-W4P variants hydrodynamically have shown active virion production, HBsAg secretion, and high levels of IL-6 [17, 18]. This* in vivo* model might be appropriate to study antiviral and anti-inflammatory activities of KCT-01. Therefore, the objective of the current study was to determine the toxicity of KCT-01 and examine its antiviral activity against HBV and its inflammation suppressing activity using* in vitro* and* in vivo* W4P variant hydrodynamic injection models.

## 2. Materials and Methods

### 2.1. Preparation of KCT-01 and ETV

KCT-01 was produced and examined by Hanpoong Pharm. Co., Ltd (Jeollabuk-do, South Korea) under conditions and practices required by Korea Good Manufacturing Practice (KGMP). Briefly, raw herbal mixture of* A. capillaris* (12 kg),* S. officinalis* (4 kg), and* C. longa* (4 kg) was purchased from Kyung Hee Herb Pharm (Wonju, South Korea), cut, and pulverized with a pulverizer (30 mm) followed by extraction with a 10-fold volume of 30% EtOH (80-90°C, 3 h). After the first filtration with 5 *μ*m microfilter, the same process of extraction and filtration was then repeated. KCT-01 (3.74-4.56 kg) of freeze-dried extract powder was prepared by vacuum evaporation of filtrates at temperature under 60°C (average extraction yield: about 20.75%). ETV was purchased from Lianyungang Guike Pharmaceutical Co., Ltd (Jiangsu, China). Its purity was 99.9% and its water content was 0.4%. It was white or offwhite powder in appearance.

### 2.2. Analysis of KCT-01

Curcumin (C_21_H_20_O_6_) and 6,7-dimethylesculetin (98%, C_11_H_10_O_4_) were purchased from Sigma-Aldrich (St. Louis, MO, USA). Ziyuglycoside I (98%, C_41_H_66_O_13_) was purchased from Wuhan ChemFaces Biochemical Company (Hubei, China). To set reference content of these three compounds in KCT-01, test solutions of* A. capillaris*,* S. officinalis*, and* C. longa* were produced through a series of processes of weighing, methanol addition, extraction, and filtration. To prepare standard solutions, 6,7-dimethylesculetin, ziyuglycoside I, and curcumin were diluted with methanol solution. All test solutions and standard solutions (10 *μ*L each) were subjected to liquid chromatography. Their peak areas were measured under established conditions for standardization.

### 2.3. Cells

HepG2 (ATCC HB-8065) and HepG2.2.15 human hepatocellular carcinoma cells [19] were maintained in Dulbecco's modified Eagle's medium (DMEM) containing 10% fetal bovine serum (FBS) and penicillin/streptomycin (100 U/ml).

### 2.4. Cell Cytotoxicity Assay

Effects of KCT-01 on cell viability were determined by 3-(4,5-dimethylthiazol-2-yl)-2,5-diphenyltetrazolium bromide (MTT) assay. Briefly, HepG2 and HepG2.2.15 cells were treated with increasing concentrations of KCT-01 for 24 h and 48 h. After further incubation with MTT-containing medium for 4 hours, cells were lysed with dimethyl sulfoxide. Absorbance of cell lysate was determined at wavelength of 570 nm using a microplate reader (BMG LABTECH, Ortenberg, Germany).

### 2.5. In Vivo Toxicology Study

Preclinical toxicology test of KCT-01 was performed in ChemOn Inc (Gyeonggi-Do, South Korea), a private contract research organization (CRO) in Korea for nonclinical area certified with Korea Good Laboratory Practice (KGLP). Single dose toxicity and repeated dose toxicity studies were performed according to “Guidelines for toxicity tests for drugs and related materials, #2015-82” and “Guidelines for preclinical trials of herbal medicine, #B1-2014-3-014” prepared by the Korean Ministry of Food and Drug Safety. A total of 40 Crl:CD SD rats of each sex (males: 20, females: 20) at 8 weeks old were divided into four groups (KCT-01 1250, 2500, and 5000 mg/kg/day groups and a vehicle control group) to evaluate the approximate lethal dose (ALD) in the single dose toxicity test (study no. 17-RA-0037). For a 2-week-repeated-oral-dose study of KCT-01, a total of 80 healthy rats were randomly assigned into four groups (KCT-01 1250, 2500, and 5000 mg/kg/day groups, and a vehicle control group, and 20 rats (males: 10, females: 10) per group). Clinical signs, serum/urine analysis, and histological analysis were performed to evaluate no-observed-adverse-effect level (NOAEL) (study no. 17-RR-0038).

### 2.6. Analysis of HBsAg and HBeAg Secretion by Enzyme-Linked Immunosorbent Assay (ELISA)

HepG2.2.15 cells were treated with ETV or KCT-01 to detect the secretion of HBsAg and HBeAg for 24 h and 48 h, respectively. Levels of HBsAg and HBeAg secreted from HepG2.2.15 cells were determined by ELISA using HBsAg detection kits (Shanghai Shiye Kehua Company, Shanghai, China) and HBeAg detection kits (DIAsource ImmunoAssays SA, Louvain-la-Neuve, Belgium) according to manufacturers' instructions.

### 2.7. Determination of Virion Particle Production and Viral Pregenomic RNA (pgRNA) Levels by Quantitative PCR

HepG2.2.15 cells were treated with ETV or KCT-01 for 48 h. HBV DNAs and total pgRNAs were extracted from culture supernatants using Viral Gene-Spin Kit (Intron, Gyeonggi-do, South Korea) and RNA extraction Kit (Geneall, Seoul, South Korea) according to manufacturers' instructions, respectively. PCR amplifications were performed using a set of real-time PCR primers listed in Table 1.

### 2.8. Animals

C57BL/6 male mice (Specific-pathogen-free, 8-week old, 20-25 g) were obtained from Orientalbio (Seoul, South Korea). These animals were cared under standard conditions with water and food provided ad libitum following NIH guidelines for housing and care of laboratory animals in the Animal Facility of College of Medicine, Seoul National University (Seoul, South Korea). All animal experiments were conducted following the protocol approved by Institutional Animal Care and Use Committee (IAUAC) of Seoul National University College of Medicine (protocol no. SNU150609).

### 2.9. Hydrodynamic Injection

To determine* in vivo* antiviral activities of KCT-01 against HBV, hydrodynamic injection of pHBV-1.2X-W4P harboring HBV variant full genome was performed. For systemic hydrodynamic injection of pHBV-1.2X-W4P /HBV* in vivo*, a total of 10 *µ*g of plasmids in PBS was injected into the tail vein within 5–8 seconds. The injected volume was 8% of the mouse's body mass (e.g., 1.6 ml for a mouse of 20 g). Mice were housed in a biohazard facility until sacrifice. A reporter plasmid pIRES2-*Luci* expressing* Renilla luciferase* was also included in the injection mixture as an internal control to evaluate the delivering efficiency.

### 2.10. Determination of In Vivo Anti-HBV Activity of KCT-01

To evaluate* in vivo* antiviral effects, ETV and KCT-01 were administered to mice injected with HBV genome hydrodynamically. Various dosages of ETV and KCT-01 were delivered into the stomach using an oral gavage needle in a volume of 0.1 ml. At 24 h after hydrodynamic injection, mice were administered with ETV at 0.2 mg/kg or KCT-01 at 250 or 500 mg/kg once daily for 14 days. Two other groups were administered with both ETV (0.2 mg/kg) and KCT-01 (250 or 500 mg/kg) to test the effect of coadministration. PBS was used as control. At 14 days after administration, blood samples were collected and sera were stored at −20°C before use. These mice were sacrificed and samples of liver tissues were collected. At least five mice were included in each group. HBV DNA was extracted from 30 *μ*l of mouse serum sample collected at 14 days after administration and analyzed by qPCR as described in Section 2.7. Levels of covalently closed circular (ccc) DNA in liver tissue samples were quantified using a set of specific primers (Table 1) to amplify and detect cccDNA. Briefly, liver tissues were lysed with lysis buffer A (50 mM Tris-HCl, pH7.4, 1 mL EDTA, and 1% NP-40) and subjected to sonication (on ice, 1-2 pulses of 5 sec), centrifugation (10,000×g, 25°C, 1 min), resuspension (lysis buffer B (10 mM Tris-HCl, 10 mM EDTA, 150 mM NaCl, 0.5% sodium dodecyl sulfate, and 0.5 mg/mL proteinase K), and incubation (37°C, 24 h). After purification using MEGA Quick Spin Kit, cccDNA was quantified by qPCR.

### 2.11. Isolation and Analysis of Mouse Liver Tissue mRNAs

Total RNAs were extracted from liver tissues using TRIzol Reagent (Life Technologies, Carlsbad, CA, USA). They were treated with ribonuclease-free deoxyribonuclease I (Roche, Mannheim, Germany) for 40 min at 37°C to eliminate residual DNA followed by phenol/chloroform extraction, ethanol precipitation, and resuspension with diethyl pyrocarbonate–treated water. Extracted RNAs from liver tissues were then subjected to reverse transcription and subsequent PCR reaction. The values were normalized to those of 18S rRNA. Primers used for qPCR are listed in Table 1. Cycling parameters were as follows: 1 cycle of 45°C for 10 min, 1 cycle of 95°C for 2 min, and 40 cycles of 95°C for 5 s and 60°C for 20 s.

### 2.12. Statistical Analysis

Both in vitro and* in vivo* data in this study are expressed as mean ± S.D. and analyzed using Student's* t*-test with GraphPad Prism 5.01 software (GraphPad Software Inc., San Diego, CA, USA). Statistical significance was considered when* p *value was below 0.05.

## 3. Results and Discussion

### 3.1. Preparation and Standardization of KCT-01

To set criteria for contents of three major reference constituents (6,7-dimethylesculetin, ziyuglycoside I, and curcumin) from 1 g of KCT-01, dry material of KCT-01 manufactured in three LOTs (16001, 16002, and 16003) was repeatedly tested (three times) to obtain the content of each index component in a KGMP certified company. Values of over 90% of the average content of each compound were set as criteria for standardization. As shown in Table 2, quantitative analysis of KCT-01 revealed that 1.76 mg/g of 6,7-dimethylesculetin, 21.09 mg/g of ziyuglycoside I, and 0.61 mg/g of curcumin or more should be included in KCT-01 extract.

### 3.2. In Vitro and In Vivo Toxicological Study of KCT-01

**Figure 1:**
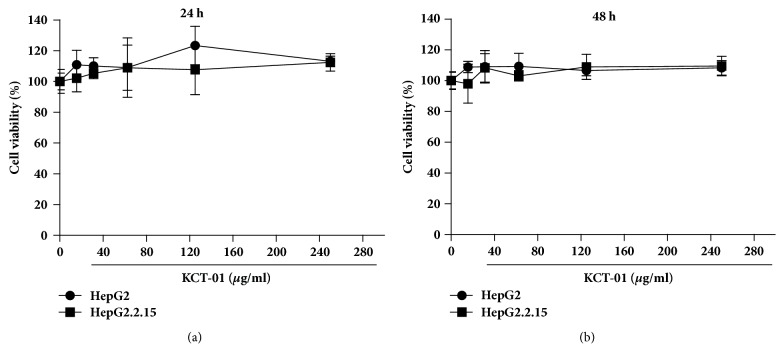
Analysis of* in vitro* cell cytotoxicity of KCT-01 on HepG2 and HepG2.2.15 cells. (a-b) HepG2 and HepG2.2.15 cells were treated with increasing doses of KCT-01 for 24 h (a) or 48 h (b). Cell viability was determined by MTT assays. Error bars represent standard deviations from triplicated samples.

*In vitro* cell cytotoxicity of KCT-01 was evaluated using HepG2 cells and HepG2.2.15 cells by MTT assays. As shown in Figures 1(a) and 1(b), treatment with KCT-01 at concentration up to 250 *μ*g/ml for 24 or 48 hours did not induce any significant cell cytotoxicity of HepG2 or HepG2.2.15 cells.

For* in vivo* preclinical safety evaluation of KCT-01, single-dose and 2-week repeated-dose toxicity studies were performed by a CRO (ChemOn Inc., South Korea) in accordance with Korea Good Laboratory Practice (KGLP). No deaths were observed in either study (single-dose study and repeated-dose study) during the observation period for two weeks. Consistent with results from the single-dose oral toxicity study, no toxic influence of KCT-01 administration (1250, 2500, and 5000 mg/kg/day) was observed based on results of body weight, food intake, hematological examination, autopsy findings, and histopathological examination in the 2-week repeated-dose study. Test substance-colored stool, drooling, reduced water intake, increased ketone, protein, and pH in urine analysis, decreased urine volume, increased albumin/globulin ratio and K^+^ levels in biochemistry analysis were observed during the 2-week administration period. However, these occurrences were judged to be caused by the influence of the test substance. They were not toxicologically harmful changes based on histopathological results. As a result of a single oral administration of KCT-01, ALD was found to exceed 5000 mg/kg/day regardless of sex. NOAEL of KCT-01 was determined to be up to 5000 mg/kg/day in both female and male rats.

### 3.3. Suppression of HBsAg and HBeAg Production in HepG2.2.15 Cells by KCT-01

**Figure 2:**
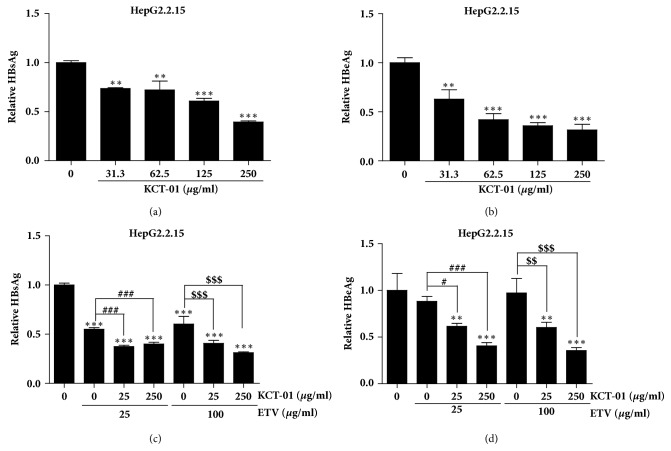
Suppression of HBsAg and HBeAg secretion by KCT-01 in HepG2.2.15 cells. (a-b) HepG2.2.15 cells were treated with increasing amounts of KCT-01 for 48 h. Amounts of HBsAg (a) and HBeAg (b) in culture supernatants were analyzed by ELISA. (c-d) HepG2.2.15 cells were treated with different concentrations of KCT-01 with ETV. Secreted amounts of HBsAg (c) and HBeAg (d) were analyzed by ELISA. Values are provided as mean ± S.D. of triplicated experiments. Statistical significance was processed using Student's* t*-test. ^*∗∗*^*p* < 0.01 and ^*∗∗∗*^*p* < 0.001 compared to control conditions; ^#^*p* < 0.05 and ^###^*p* < 0.001 compared to ETV (25 *μ*g/ml-) treated group; ^$$^*p* < 0.01 and ^$$$^*p* < 0.001 compared to ETV (100 *μ*g/ml-) treated group.

The three herbal medicines (*A. capillaris*,* S. officinalis*, and* C. longa*) as components of KCT-01 have been implicated to have efficacy for HBV-related liver diseases and hepatitis. To examine the antiviral function of KCT-01 against HBV, its ability to suppress secretion of HBsAg and HBeAg in HepG2.2.15 stably transfected with HBV genome was determined. Treatment with KCT-01 resulted in decreased secretion of HBsAg and HBeAg in HepG2.2.15 cells in a dose-dependent manner (Figures 2(a) and 2(b)). KCT-01 at 250 *μ*g/ml was sufficient to reduce secretion of both HBsAg and HBeAg to less than 50% compared to mock-treated control. KCT-01 at 62.5 *μ*g/ml was also able to suppress secretion of HBeAg to less than 50% of the control. These results demonstrate that KCT-01 can inhibit the production of HBV antigens from HBV genomes existing in cells.

Next, we examined the effect of KCT-01 on secretion of HBV antigens when it was cotreated with ETV. As depicted in Figure 2(c), 25 *μ*g/ml of KCT-01 reduced the secretion of HBsAg to approximately 40% of section in mock-treated control when it was treated together with ETV. Suppressive effect of ETV on HBsAg secretion was enhanced by cotreatment with KCT-01 in a dose-dependent manner (Figure 2(c)). These results indicate that KCT-01 cotreatment with ETV does not interfere with the action of ETV. Antiviral effects of KCT-01 and ETV can be augmented by such combined treatment. In case of HBeAg, ETV alone did not induce significant reduction of HBeAg. Cotreatment and KCT-01 treatment alone showed similar results (Figure 2(d)). Since ETV exerts its antiviral activity through inhibiting HBV replication, it may not be able to suppress HBeAg production from HepG2.2.15 cells stably presenting the HBV genome. The discrepancy between effects of ETV on HBsAg and HBeAg production in HepG2.2.15 cells remains elusive. Nonetheless, the suppressive effect of KCT-01 on HBeAg production was not affected by ETV, suggesting that combined treatment of KCT-01 and ETV might be used as a possible therapeutic strategy against HBV chronic infection.

### 3.4. Inhibition of HBV Virion Particle Production and pgRNA Synthesis by KCT-01

**Figure 3:**
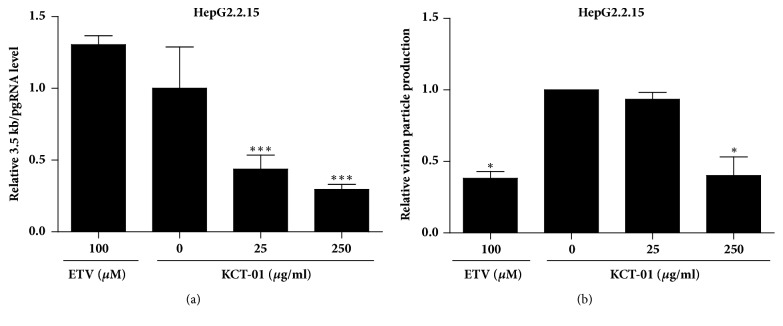
Suppression of virion particle production and pgRNA synthesis by KCT-01 in HepG2.2.15 cells. (a-b) HepG2.2.15 cells were treated with KCT-01 (0, 25, or 250 *μ*g/ml) or ETV (100 *μ*M) for 48 h. Levels of 3.5 kb pgRNA (a) and viral DNAs (b) were quantified by qPCR. Values are provided as mean ± S.D.s of triplicated experiments. Statistical significance was processed using Student's* t*-test. ^*∗*^*p* < 0.05 and ^*∗∗∗*^*p* < 0.001 compared to ETV-treated group.

To further confirm the antiviral activity of KCT-01, its effect on viral pgRNA production in HepG2.2.15 cells was examined. As shown in Figure 3(a), 3.5 kb pgRNA levels in HepG2.2.15 cells treated by KCT-01 at 25 and 250 *μ*g/ml were significantly (*p* < 0.001) reduced compared to those in the control group in a dose-dependent manner. However, they were not significantly affected by ETV. Thus, KCT-01 might exert its antiviral activity against HBV through mechanism distinct from ETV. The reduction of pgRNA synthesis by KCT-01 indicates that KCT-01 is capable of suppressing viral replication and virion production of HBV. Indeed, levels of virion particles in culture supernatants of KCT-01-treated HepG2.2.15 cells were reduced. Treatment with 250 *μ*g/ml of KCT-01 for 48 h resulted in significant (*p* < 0.05) reduction of virion particle production in HepG2.2.15 cells. Such effect was comparable to that of treatment with 100 *μ*M ETV (Figure 3(b)). These results indicate that the antiviral effect of KCT-01 is not only due to suppression of viral antigen production, but also due to suppression of viral particle production.

### 3.5. In Vivo Anti-HBV Activity and Anti-Inflammatory Activity of KCT-01

**Figure 4:**
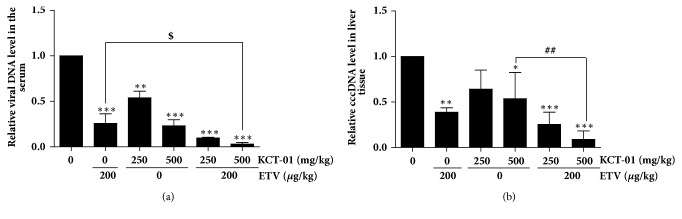
*In vivo* antiviral activity of KCT-01 against HBV. (a-b) Levels of serum HBV DNA (a) and cccDNA from liver tissues (b) were quantified by qPCR assay. Values are provided as mean ± S.D. of triplicated experiments. Statistical significance was processed using Student's* t*-test. ^*∗*^*p* < 0.05, ^*∗∗*^*p* < 0.01, and ^*∗∗∗*^*p* < 0.001 compared to control conditions; ^$^*p* < 0.05 compared to ETV single administration group; ^##^*p* < 0.01 compared to KCT-01 single administration group.

To further confirm the antiviral role of KCT-01, its activity was examined using a mouse hydrodynamic injection model. Mice were injected with HBV full genome and orally administered with KCT-01 alone or in combination with ETV for 14 days. Previously, we have reported that HBV variant is implicated in increased risk of progression of liver diseases [17, 18]. Thus, we injected HBV-full-genome with W4P mutation as described in Materials and Methods [19]. As depicted in Figure 4(a), administration of KCT-01 reduced viral DNA levels in mice sera in a dose-dependent manner, indicating that KCT-01 effectively inhibited* in vivo* HBV virion production. In addition, suppression of HBV production by ETV was significantly augmented by coadministration with KCT-01.

Next, we examined levels of intracellular cccDNA in liver tissues. In line with previous results, both KCT-01 at 500 *μ*g/kg and ETV at 200 *μ*g/kg administration significantly reduced levels of cccDNA extracted from liver tissues compared to the control group. In particular, concurrent administration of ETV and KCT-01 exhibited more potent reduction in cccDNA levels than single administration of either ETV or KCT-01 (Figure 4(b)). Inhibition of cccDNA formation is an important mechanism for treating CHB because current antiviral strategies have some limitations to completely eradicate cccDNA. These results suggest that KCT-01 might have potential as a novel therapeutic agent against HBV infection because its antiviral action could affect cccDNA formation, the early stage of viral replication.

**Figure 5:**
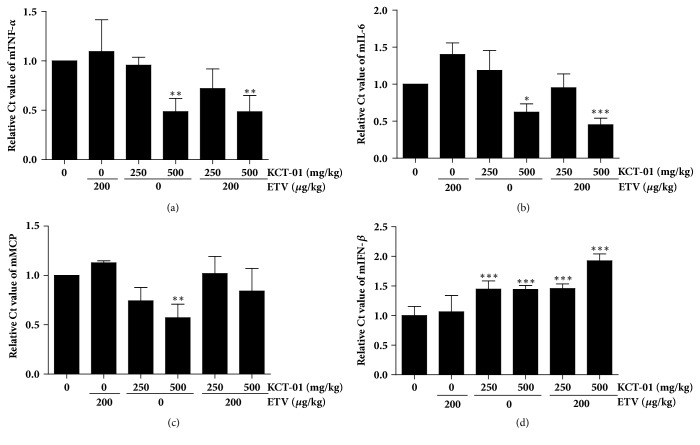
Suppression of HBV-mediated inflammatory responses by KCT-01 administration. (a-d) mRNA levels of TNF-*α* (a), IL-6 (b), MCP (c), and IFN-*β* (d) in liver tissues from mice injected with HBV genome were analyzed by qPCR assay. Values are provided as mean ± S.D. of triplicated experiments. Statistical significance was processed using Student's* t*-test. ^*∗*^*p* < 0.05, ^*∗∗*^*p* < 0.01, and ^*∗∗∗*^*p* < 0.001 compared to control conditions.

Chronic inflammatory response caused by HBV chronic infection is the main risk factor for liver cirrhosis and HCC [20]. Thus, we also examined the effect of KCT-01 on HBV-mediated inflammatory responses using liver tissues from the hydrodynamic injection model. Notably, mice liver tissues from 500 *μ*g/ml of KCT-01-treated group showed significant reduction in TNF-*α*, IL-6, and MCP mRNA synthesis. However, ETV treatment did not cause any significant change in mRNA levels of these genes (Figures 5(a), 5(b), and 5(c)). These results indicate that KCT-01 is capable of suppressing HBV-mediated inflammatory responses in addition to its inhibitory effect on viral replication. Considering that ETV did not display any significant effect on inflammatory cytokine or chemokine production, the effect of KCT-01 on inflammatory cytokine and chemokine production was not due to its suppression of viral replication. Presumably, KCT-01 itself might have potent anti-inflammatory activities. This is supported by a preclinical* in vitro* and* in vivo* study showing that KCT-01 can regulate inflammatory cytokines via suppression of Janus kinase [21]/signal transducer and activator of transcription (STAT) signaling pathway [22]. Interestingly, interferon (IFN)-*β* production was not suppressed by KCT-01 administration (Figure 5(d)). In fact, it was increased by KCT-01. This indicates that IFN-*β* mediated innate antiviral immune response is not hampered by KCT-01, unlike other inflammatory cytokine responses. The underlying molecular mechanism remains elusive.

HBV is known to be a noncytopathogenic virus which replication itself does not cause direct hepatocellular toxicity, but its ongoing attack to hepatocytes involves variable inflammatory cytokines secretion, eventually contributing to the development of cirrhosis and cancer [23]. Therefore, regulating inflammatory cytokines play an important role in treating CHB as well as inhibiting HBV DNA polymerase replication. However, currently validated antivirals have little capacity in modulating inflammation and eradicating HBV DNA. In addition, some adverse effects including lactic acidosis, disease progression, low HBeAg seroconversion, and drug resistance have been emerged as new issues during antiviral agents therapy [24, 25]. Therefore, the antiviral benefits of KCT-01 including suppressions of cccDNA formation, inflammatory cytokines, and antigen secretion by HBV might help physicians to apply optimal therapeutic strategies to manage CHB patients and it could be a novel potent antiviral candidate as not only single treatment but also cotreatment with conventional antivirals without hampering the efficacy of ETV.

## 4. Conclusions

This study demonstrates that KCT-01, a novel herbal decoction manufactured and examined according to KGMP and KGLP, exerts robust antiviral activities against HBV using* in vitro* and* in vivo* systems. Anti-HBV activity of ETV, a clinically proven therapeutic agent, can be significantly enhanced by KCT-01, suggesting that a combination of the two could be used as a potential therapeutic strategy. Furthermore, KCT-01, but not ETV, possesses anti-inflammatory activity. Their combination can suppress both virus replication and virus induced hepatitis. Significant suppression of IL-6 mRNA levels by KCT-01 was increased in the liver of W4P variant hydrodynamic injection mice, strongly indicating its preventive role in the progression of liver cirrhosis or HCC. Taken together, our results indicate that KCT-01 can efficiently regulate inflammatory cytokines such as IL-6 and inhibit tumorigenesis of HCC in addition to its suppression effect on the virus. Our findings may partly provide preclinical evidence to develop KCT-01 as an effective therapeutic agent to treat CHB and prevent HCC not only by itself alone, but also in combination with other antiviral agents.

## Figures and Tables

**Table 1:** Primers used to analyze mice liver tissue mRNAs.

		Forward	Reverse	Remarks
**1**	**mTNF-**α	AGGGTCTGGGCC ATAGAACT	CCACCACGCTCTT CTGTCTAC	

**2**	**mIL-6**	GACAACTTTGGC ATTGTGG	ATGCAGGGATGA TGTTCTG	

**3**	**mMCP**	ATTGGGATCATCT TGCTGGT	CCTGCTGTTCACA GTTGCC	

**4**	**mIFN-**β	AGCTCCAAGAAA GGACGAAC	GCCCTGTAGGTG AGGTTGAT	

**5**	**3.5 kb/pg RNA**	GGTCCCCTAGAA GAAGAACTCCCT	CATTGAGATTCCC GAGATTGAGAT	

**6**	**cccDNA level **	CCGTCTGTGCCTT CTCAT	CACAGCTTGGAG GCTTGAAC	PROBE: CGTGTGCACTTCG CTTCACCTCTGC

**7**	**small S**	TTGACAAGAATC CTCACAATACC	GGAGGTTGGGGA CTGCGAAT	Real-SF (positions 218-240) Real-SR (positions 309-328)

**8**	**HBV virion particle **	TTAACAAGAATC CTCACAATA	GGAGGTTGGGGA CTGCGAAT	

**9**	**18S rRNA**	AGTCCCTGCCCTT TGTACACA	CGATCCGAGGGC CTCACTA	

**Table 2:** Contents of three reference constituents from KCT-01.

Compounds	6,7-Dimethylesculetin			Ziyuglycoside I			Curcumin		
**LOT 16001**	1.93	1.93	1.91	23.07	22.91	23.07	0.67	0.67	0.67
**LOT 16002**	2.00	1.97	1.98	24.56	24.53	24.80	0.71	0.67	0.67
**LOT 16003**	1.97	1.96	1.93	22.67	22.56	22.66	0.69	0.68	0.68
**Average (mg/g)**	1.95			23.43			0.68		
**90% of average (mg/g)**	1.76			21.09			0.61		

## Data Availability

The data that support the findings of this study are available from the corresponding author, [Lee JH, Kim BJ and Inn KS], upon reasonable request.

## Abbreviations

CHB: Chronic hepatitis B
HCC: Hepatocellular carcinoma
ETV: Entecavir
DMEM: Dulbecco's modified Eagle's medium
FBS: Fetal bovine serum
MTT: 3-(4,5-dimethylthiazol-2-yl)-2,5-diphenyltetrazolium bromide
CRO: Contract research organization
ALD: Approximate lethal dose
NOAEL: No-observed-adverse-effect level
ELISA: Enzyme-Linked Immunosorbent Assay
pgRNA: Pregenomic RNA
cccDNA: Covalently closed circular DNA.
